# Acupuncture for irritable bowel syndrome: study protocol for a multicenter randomized controlled trial

**DOI:** 10.1186/s13063-018-2922-y

**Published:** 2018-10-01

**Authors:** Li-xia Pei, Hao Geng, Hao Chen, Xiao-liang Wu, Lu Chen, Jun-ling Zhou, Lu Ju, Gao Lu, Wan-li Xu, Shan Qin, Jing Guo, Eun Mee Yang, Jian-hua Sun

**Affiliations:** 1 Department of Acupuncture, Jiangsu Province Hospital of Traditional Chinese Medicine, Affiliated Hospital of Nanjing University of Chinese Medicine, No 155, Hanzhong Road, Qinhuai District, Nanjing, 210029 China; 2 Nanjing University of Chinese Medicine, No 138, XianlinRoad, Qixia District, Nanjing, 210000 China

**Keywords:** Acupuncture, Irritable bowel syndrome, Acupuncture effect, Randomized controlled trials, Protocol

## Abstract

**Background:**

Irritable bowel syndrome (IBS) is a chronic gastrointestinal disorder characterized by abdominal pain and change of bowel habit without organic disease. A global perspective given by the World Gastroenterology Organization (WGO) points out that IBS can impact the quality of an individual’s daily life, cause socioeconomic problems and potentially impair the patient-physician relationship. It remains a problem to treat IBS due to the complicated pathophysiology. Acupuncture is an alternative therapy recommended for IBS. The aim of this study is to investigate the efficacy and safety of acupuncture therapy for patients with IBS. We also want to explore the correlation between IBS-gene subtypes and acupuncture effect.

**Methods/design:**

A multicenter randomized controlled trial will be performed in seven hospitals. Six hundred participants will be stratified into two strata (IBS-C or IBS-D). Then, patients within each stratum will be divided into an experimental group and a control group randomly. The experimental group is treated with acupuncture while the control group is treated with Western medicine. All the patients will receive a 6-week treatment and a 3-month follow-up. The primary outcome is the IBS-Symptom Severity Score (IBS-SSS), the secondary outcome is the score of the IBS-Quality of Life (IBS-QoL).The correlation between IBS-gene subtypes and acupuncture effect will be detected based on polymerase chain reaction-restriction fragment length polymorphism (PCR-RFLP). Outcome measures (including primary and secondary outcome measures) are collected at baseline,1 week, 2 weeks, 4 weeks, and 6 weeks of the intervention, and 12 weeks after the intervention.

**Discussion:**

This is a multicenter randomized controlled trial for IBS in China. It may clarify the efficacy of acupuncture as an alternative therapy for IBS. This is the first time ever that the potential mechanism of IBS based on genomics has been investigated.

**Trial registration:**

Chinese Clinical Trials Register, ID: ChiCTR-IOR-15006259. First registered on 14 April 2015.

**Electronic supplementary material:**

The online version of this article (10.1186/s13063-018-2922-y) contains supplementary material, which is available to authorized users.

## Background

Irritable bowel syndrome (IBS) is the most common functional gastrointestinal disorder, yet it is also one of the least well-understood medical conditions. Epidemiological data shows that the prevalence of IBS is approximately 11% in the world [1] and 5–6% in China [2]. The main clinical feature of IBS is abdominal pain related to defecation or a change in stool form and/or frequency at least once a week in the preceding month [3, 4]. Although IBS is not a life-threatening disorder, it significantly reduces the patient’s quality of life and imposes a considerable economic burden on individuals, families, and society, with decreased work productivity and increased healthcare spending and utilization [5, 6].

Abnormalities in the brain-gut interaction and gastrointestinal mobility, and visceral hypersensitivity are the main pathophysiological basis of IBS, but their underlying mechanisms have not been fully elucidated. IBS is also associated with various psychosocial and environmental factors, including early life stress, food intolerance, antibiotic abuse, and intestinal infections. In addition, family and twin aggregate studies have suggested the role of genetic factors in the pathogenesis of IBS [7, 8]. In particular, polymorphisms in the serotonin reuptake transporter (*SERT*) gene have been widely implicated in the disturbances of gastrointestinal functions in IBS. The *SERT* gene is responsible for controlling the synaptic concentration of serotonin, or 5-hydroxytryptamine (5-HT), which is an important neurotransmitter and paracrine signaling molecule involved in the brain-gut axis and regulation of gastrointestinal mobility and sensation. So, modifications in the *SERT* gene affect the intensity and duration of serotonergic signaling. Recent studies have identified several polymorphisms in the serotonin-related genes associated with IBS, including the 5-HT-transporter-gene-linked polymorphic region (*5-HTTLPR*), variable number of tandem repeats *STin2*, and the single-nucleotide polymorphism (SNP) *rs25531* [9–12].

Despite these findings and advances in IBS research, the pathophysiology of IBS is still poorly understood with no definitive biomarker, thereby hampering the development of effective therapies for IBS [13]. Currently available treatments for IBS can be broadly classified into pharmacological therapies and other interventions, such as dietary and lifestyle modifications [6, 14]. There are various pharmacological treatments for IBS, including 5-HT-receptor modulating agents, antispasmodics, probiotics, and antidiarrheal agents [15]. While some have been found to be effective, they are not uniformly effective across all patients. There are also potential risks of adverse effects and substantial financial burden associated with the use of pharmacological treatments [6, 16].

As a result of dissatisfaction with the effectiveness, safety, and costs of pharmacological treatments, there has been a rise in the use of non-pharmacological therapies, including acupuncture [17]. Acupuncture is an important modality of traditional Chinese medicine (TCM), which involves an insertion of a needle into the body at specific points to prevent and treat various diseases. It has a long history originating in ancient China and has been used for thousands of years to treat digestive problems. In recent research, there has been a growing interest in investigating the possible role of acupuncture in improving symptoms and quality of life in IBS patients [18]. Current evidence suggests that acupuncture is more effective than pharmacological therapies [19]. However, there are still limitations with current research, including small sample sizes and lack of detailed assessment of improvement and side effects [20]. In addition, the role of genetic polymorphisms in the treatment of IBS with acupuncture has not yet been studied. In 2014, our team conducted a meta analysis of IBS research in seven countries, and showed that there was a correlation between the 5-HTTLPR gene polymorphism and the incidence of IBS-D and IBS-C [21, 22]. As a result, large-scale, methodologically rigorous studies are needed to assess the effectiveness and safety of acupuncture for IBS as well as investigate the role of genetics in IBS.

## Methods/design

The protocol for this trial is reported based on the Standard Protocol Items: Recommendations for Interventional Trials (SPIRIT) 2013 Checklist: defining standard protocol items for clinical trials (Additional file 1). The study has been approved by the Ethics Committee of Jiangsu Province Hospital of TCM (the Approved No. of the Ethics Committee is 2015NL-030-02) and was registered on at the Chinese Clinical Trial Registry (Chictr) platform on 14 April 2015 (Registration number: ChiCTR-IOR-15006259).

### Study design

This study is a multicenter, stratified randomized controlled trial (RCT), comparing acupuncture to standard pharmacological treatment for two subtypes of IBS: IBS-C (constipation-predominant IBS) and IBS-D (diarrhea-predominant IBS). The study is being conducted in the following seven hospitals: Jiangsu Hospital of TCM, Wuxi Hospital of TCM, Nantong Hospital of TCM, Nanjing Hospital of Chinese Medicine, Zhenjiang Riverside Hospital, Kunshan Hospital of TCM, and Shuyang Hospital of TCM. The study will be carried out according to the principles of the Declaration of Helsinki (version Edinburgh 2000).

**Fig. 1:**
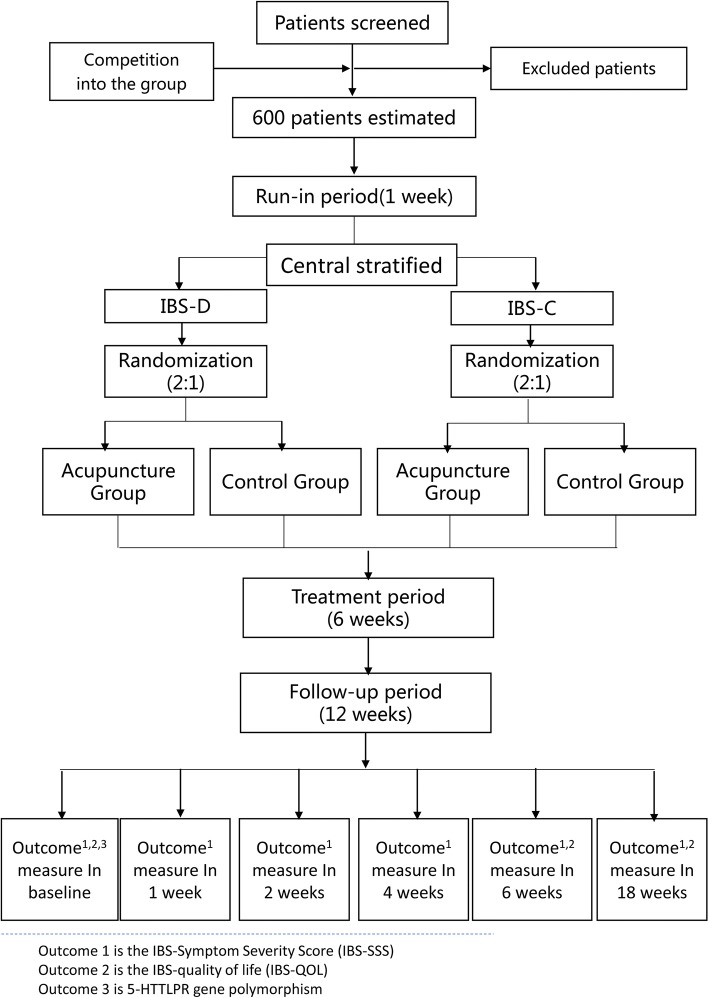
Trial flow chart

**Fig. 2:**
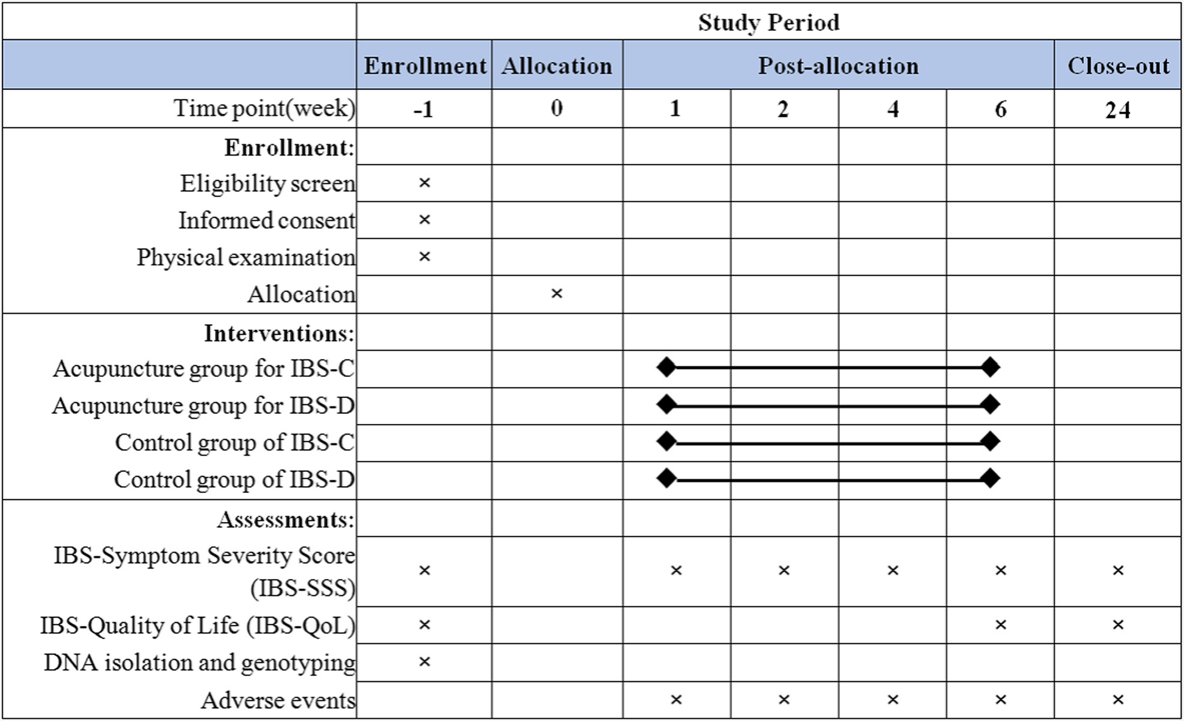
Standard Protocol Items: Recommendations for Interventional Trials (SPIRIT) Schedule for enrollment, interventions, and assessments

This study will be performed over a period of 19 weeks: 1 week of wash-out, 6 weeks of treatment, and 12 weeks of post-treatment follow-up. All eligible patients will be stratified into two strata: IBS-C or IBS-D. The patients within each stratum will then randomly divided to either an acupuncture group or a control group receiving standard pharmacological treatment. Outcomes will be assessed at baseline, during the treatment and at the end of the follow-up. Figures 1 and 2 illustrate the time schedule of enrollment, interventions, assessments, and participant visits.

### Randomization and blinding

The Good Clinical Practice (GCP) Center of Jiangsu Province Hospital of TCM will be responsible for generating the randomization sequence. All eligible participants will be told not to take any treatment for IBS for 1 week (the wash-out period). After the wash-out period, all the participants will be stratified into IBS-C or IBS-D subgroups and then randomly allocated to either the acupuncture group or the control group by a central randomization system. An allocation ratio of 2:1 will be used in the acupuncture to control group assignment. Only the outcome assessors and the statisticians will be blinded to the allocation. The outcome assessors and the statisticians will not participant in the treatment, they will perform the outcome evaluation and the statistical analysis independently.

### Study participants and the recruitment

Participants will be recruited from the outpatient centers of the seven participating hospitals and their surrounding communities by posting and distributing posters and flyers about this trial.

The following inclusion criteria will be used: (1) adults aged 18–70 years, (2) diagnosis of IBS-C or IBS-D, as defined by ROME III diagnostic criteria [4], (3) IBS duration of 6 months or longer, (4) lack of morphological changes and biochemical abnormalities that could explain the symptoms, (5) baseline IBS-Symptom Severity Score (IBS-SSS) score of 75 points or greater, (6) no acupuncture treatment for 3 months preceding the trial, (7) no participation in any other ongoing clinical trial, and (8) agreement to participate and signing of the informed consent document.

The exclusion criteria are: (1) diagnosis of an organic intestinal disease, (2) history of abdominal and/or rectal surgery, (3) history of drug dependence for gastrointestinal motility and function, (4) other serious medical conditions, such as cardiovascular disease, endocrine disorders, hepatic dysfunction, cerebral vascular disease, renal diseases, and cognitive disorders, and (5) pregnancy, lactation, and postpartum period of less than 12 months. Physical examination will be conducted at the time of recruitment to exclude participants with serious diseases not suitable for this trial.

### Acupuncture and the control group

**Fig. 3:**
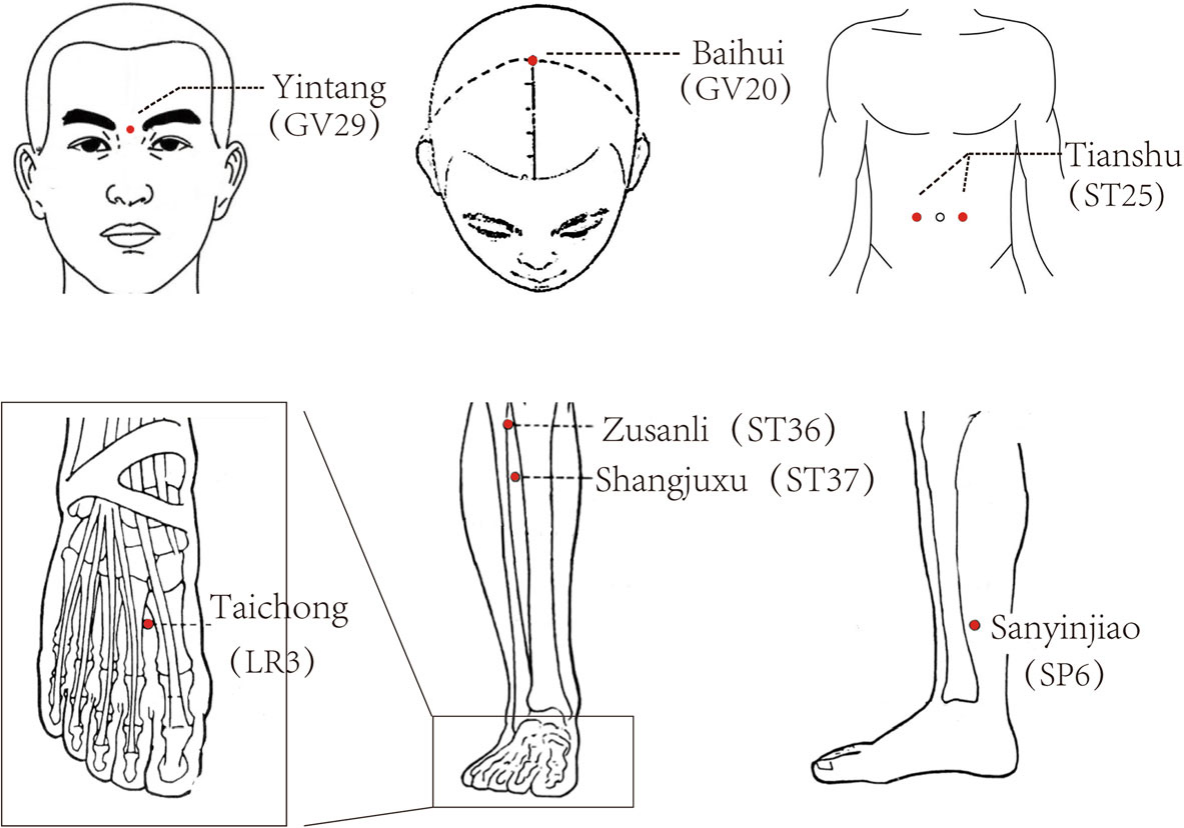
Locations of acupoints: acupuncture group

Patients in the acupuncture group, regardless of the IBS subtypes, will receive acupuncture three times a week for 6 weeks for a total of 18 treatment sessions. Patients will receive treatment in supine position for 30 min per session. The treatment will be provided by licensed acupuncturists holding acupuncture physician certifications in China with at least 2 years of clinical experience. Disposable, sterile needles with a diameter of 0.30 mm and a body length of 40 mm (Huatuo, Suzhou, China) will be used. Based on TCM theory and our clinical experience, acupoints used here are unilateral *GV20* (*Bai Hui*), *GV29* (*Yin Tang*) and bilateral *LR3* (*Tai Chong*), *ST36* (*Zu San Li*), *SP6* (*San Yin Jiao*), *ST25* (*Tian Shu*), and *ST37* (*Shang JuXu*) (Fig. 3). The exact location and depth of needling for each point will be determined based on the 2006 People’s Republic of China National Standard (GB/T 12346–2006) Acu-Points Name and Location [23]. After insertion, all points will be manually stimulated by lifting and thrusting the needle every 10 min to elicit the “*deqi*” and the needles will be retained for 30 min.

The patients in the control group will receive standard pharmacological treatment based on the IBS subtype. Patients with IBS-C will take macrogol 4000 powders (Fu Song, two bags per day, dissolved in a glass of water before taking) for 6 weeks. Patients with IBS-D will take pinaverium bromide tablets (Dicetel, 50 mg tid, swallowing pills with water during meals without breaking, chewing or dissolving) for 6 weeks.

### Outcome assessment

The primary outcome measure of this trial is the IBS-Symptom Severity Score (IBS-SSS), a validated scoring system for assessing the severity of IBS [24, 25]. The questionnaire is scored from 0 to 500 and is composed of five 100-point scales that measure the severity of pain, duration of pain, severity of abdominal distention, dissatisfaction with bowel habits, and disruption in life quality. The scores are classified into no IBS (< 75), mild IBS case (75–175), moderate IBS case (175–300), and severe IBS case (> 300) [25]. IBS-SSS is assessed at baseline, 1, 2, 4, 6, and 18 weeks.

The secondary outcome measures include the IBS-Quality of Life (IBS-QoL) and genotyping of the patients’ serotonin-related genes. The IBS-QoL is a 34-item questionnaire for determining the degree to which IBS interferes with the patient’s quality of life [26]. It is composed of eight dimensions, including dysphoria, interference with activity, body image, health worry, food avoidance, social reaction, sex, and relationships. Each item is evaluated on a 5-point rank scale with a higher score indicating a better quality of life. The IBS-QoL will be measured at baseline and 6 and 18 weeks.

DNA isolation and genotyping will be performed to study the possible correlation between the therapeutic effectiveness of acupuncture and the following polymorphisms of serotonin-related genes: (1) *HTTLPR* (*L/L*, *L/S*, *S/S*), (2) *STin2 VNTR* (*STin2 12/12*, *STin2 12/10*, *STin2 12/9*, *STin2 10/10*, *STin2 9/9*), and (3) *rs25531* (*rs25531-G*). The patients’ blood samples will be collected at the baseline. The correlation between IBS-gene subtypes and acupuncture effect will be detected based on cleaved amplification polymorphism sequence-tagged sites.

### Safety evaluation

During the study, adverse events (AEs) are defined as any unexpected or undesired harmful effect resulting from acupuncture or pharmacological treatment. A research assistant (GL) will be required to record all the AEs, including information on the time of occurrence, severity, duration, measurement, management, and its outcome, in a case report form (CRF). Any serious adverse events (SAEs) will be immediately reported to the principal investigator (JHS) and the Medical Ethics Committee within 24 h. The Acupuncture Department of Jiangsu Province Hospital of TCM will be responsible for the treatment of all the AEs.

### Sample size calculation and statistical analysis

Based on our previous clinical experience and the previous literature, we anticipate an improvement of 115 points in the acupuncture group and 105 points in the control group on the IBS-SSS. The standard deviation is set at 37, and the ratio between the acupuncture and control groups is 2:1. The sample size is calculated with 80% power and 5% type I error using G*Power software (version, Franz Faul, Universitat Kiel, Germany). This resulted in a required sample size of 322 participants in the acupuncture group and 161 participants in the control group. After accounting for a 20% drop-out rate, 400 participants in the acupuncture group and 200 participants in the control will be included.

Statistical analysis of all data in this study will be performed by a specialized statistician, who is blinded to the treatment allocation. Statistical Package for the Social Sciences (SPSS) V.24.0 (SPSS Inc., Chicago, IL, USA) will be used for data analysis. All analysis will be on the basis of the intention-to-treat (ITT) population and the per-protocol (PP) population. ITT requires all participants in a clinical trial to be included in the analysis in the groups to which they were randomized, regardless of any departures from randomized treatment. The PP population is usually defined as all patients completing the study without major protocol deviations – that is those who followed the rules of the study. The results of each population will be compared for stabilization. Last-observation-carried-forward will be used in the case of missing data. The principal investigator will take responsibility for the results of the data analysis. In terms of data description, quantitative and qualitative data will be presented as mean ± standard deviation, frequency, percentages or constituent ratios, respectively. Other covariates such as age, gender, ethnicity, disease duration, marital status, educational level, weight (in kilograms), and height (in meters) will be considered for further revision. In case of normality of distribution and homoscedasticity, the baseline characteristics will be performed using the analysis of covariance (ANCOVA) model, and the multiple comparisons will be performed with the Least Significant Difference test. In addition, a repeated- measure analysis of variance (rm-ANOVA) will be used to determine, within each group, the difference between baseline and 1, 2, 4, 6, and 18 weeks for both the IBS-SSS and the IBS-QoL. On this occasion of non-normal distribution data and heteroscedasticity data, the non-parametric Kruskal-Wallis test will be performed, and the Nemenyi test will be done to identify the difference between the groups. Pearson’s chi-squared test will be used in the transformed categorical data. Lastly, we will conduct rm-ANOVA to analyze genotype-related effects on treatment response. The Huynh-Feldt correction will be used if the assumption of sphericity is violated. A two-sided *p* value of less than 0.05 will be considered statistically significant for all analyses.

### Data management and monitoring

All the researchers will focus on and sign to protect the individual privacy of the participants. The raw data will be collected and cross-checked by two researchers (WLX and SQ). The database software EpiData (version 3.1) will be used for the data management. All the management will be performed in compliance with the study Standard Operation Process (SOP).

The Department of Science and Technology in Jiangsu Province Hospital of TCM, which is not taking part in the study, will be responsible for the monitoring. The CRFs, protocol compliance, data management, treatment administration and AEs will be monitored independently during the study.

## Discussion

Although IBS is a highly prevalent functional gastrointestinal disorder and has been extensively studied, there are still many unknowns regarding its pathophysiology and treatment. The currently available pharmacological treatments have limitations in terms of effectiveness, adverse effects, and costs [6, 16]. In response to these limitations, there has been a growing demand for alternative, non-pharmacological treatments for IBS, including acupuncture [27]. As an important part of TCM, acupuncture has been used in China for thousands of years to treat digestive diseases and has shown to be effective in modern clinical practice. Research evidence also suggests that acupuncture is more effective than pharmacological treatment in improving symptoms and quality of life in IBS patients. However, the evidence is still considered inconclusive or poor due to lack of methodological rigor and small sample size [19, 20]. Therefore, a large-scale multicenter RCT is needed to investigate the effect of acupuncture on IBS within the evidence-based medical framework.

The pathogenesis of IBS is still unclear, but a dysregulation of the brain-gut axis is thought to play a critical role in the disturbance of gastrointestinal function. Found in both the central nervous system and the gastrointestinal tract, serotonin is a major neurotransmitter involved in the brain-gut interaction. Elevated or reduced levels of serotonin have been implicated with diarrhea-predominant or constipation-predominant IBS, respectively [9]. The serotonin reuptake transporter (*SERT*) gene is responsible for regulating the intensity and duration of serotonergic signaling. Recent studies have shown that polymorphisms in the *SERT* gene are involved in the pathogenesis of IBS. For example, the prevalence of *5-HTTLPR l/l* and *rs25531 A/G* genotypes were found to be higher for patients with IBS compared to healthy controls [12]. Thus, there is increasing evidence for the role of genetics in the pathogenesis of IBS and growing interest in individualized treatments based on genetics studies [13]. Moreover, genetic variability may explain heterogeneity in treatment outcomes among different individuals. The effect of acupuncture on IBS symptoms may also be associated with the patients’ polymorphisms in the *SERT*-related genes. Therefore, this study will conduct genotyping of each patient to investigate the correlation between the polymorphisms of the *SERT* genes and therapeutic effect size.

From a TCM perspective, the brain-gut axis represents the interaction between the *Shen* (mind) and the Spleen. The Spleen is responsible for digesting food and generating energy for the whole body. Common manifestations of Spleen deficiency include abdominal pain, loose stools or constipation, and fatigue. Spleen deficiency is the most common syndrome associated with the diagnosis of IBS. Another important treatment principle for IBS is regulating the *Shen*. A disturbance in *Shen* or mental/emotional problems can also cause a problem in the Spleen. The close relationship between the Spleen and *Shen* is also demonstrated by studies showing the high prevalence of depression and anxiety among IBS patients compared to the healthy population. Therefore, it is important to address both the Spleen and *Shen* in treating IBS. The acupuncture points in this trial are selected in accordance with the TCM treatment principles of invigorating the Spleen and regulating the *Shen*.

Nevertheless, there are still limitations in this study that should be noted. First, the main outcome measures are subjective evaluation scales which lack objective evidence to support the effect of a given treatment directly. Second, after completion of the 6-week treatment, a 3-month follow-up will be conducted, the drop-out rate may be higher. In order to minimize potential drop out, we have established guidelines and training for research staff to establish good communication relationships with the participants.

In conclusion, the results of this methodologically rigorous trial are expected to not only provide clinical evidence of the effectiveness and safety of acupuncture in treating IBS, but also investigate the role of genetics in IBS.

### Trial status

The first participant was included on 3 May 2017 and the first version was developed on 20 May 2017. Now shown above is the third version whose protocol was revised for following reasons: unspecific statistical methods; imprecise sample size calculation; inappropriate descriptions. Till now 540 participants have been recruited. This trial is still ongoing.

## Additional file

Additional file 1: Standard Protocol Items: Recommendations for Interventional Trials (SPIRIT) 2013 Checklist. (DOC 116 kb)

## Abbreviations

5-HT: 5-hydroxytryptamine
5-HTTLPR: 5-HT-transporter-gene-linked polymorphic region
AEs: Adverse events
CRF: Case report form
GCP: Good Clinical Practice
IBS: Irritable bowel syndrome
IBS-QoL: IBS-Quality of Life
IBS-SSS: IBS-Symptom Severity Score
ITT: Intention-to-treat
PP: Per-protocol
SAEs: Serious adverse events
SERT: Serotonin reuptake transporter
SNP: Single-nucleotide polymorphism
TCM: Traditional Chinese medicine
